# Monocyte-to-lymphocyte ratio as a subtype-specific biomarker in breast cancer prognosis: a narrative review

**DOI:** 10.1097/MS9.0000000000004218

**Published:** 2025-10-29

**Authors:** Emmanuel Ifeanyi Obeagu

**Affiliations:** Department of Biomedical and Laboratory Science, Africa University, Muatre Zimbabwe

**Keywords:** biomarkers, breast cancer, monocyte-to-lymphocyte ratio, prognosis, tumor microenvironment

## Abstract

Breast cancer is a biologically diverse condition featuring unique molecular subtypes that affect prognosis and treatment response. The monocyte-to-lymphocyte ratio (MLR), obtained from peripheral blood counts, has surfaced as a potential inflammatory biomarker indicating the equilibrium between tumor-supporting monocytes and antitumor lymphocytes. This review discusses the function of MLR as a subtype-specific biomarker in breast cancer, emphasizing its potential value in forecasting disease advancement, treatment efficacy, and overall patient results. Evidence shows that increased MLR is linked to poor outcomes in various breast cancer subtypes, such as hormone receptor-positive, human epidermal growth factor receptor 2-enriched, and triple-negative breast cancers. The biological basis arises from the dual function of monocytes in promoting tumor-supportive environments and lymphocytes in facilitating immune monitoring. Through the capture of this immunological interaction, MLR acts as a low-risk and affordable method for risk assessment and treatment choices adapted to molecular subtype traits.

## Introduction

Breast cancer continues to be the most frequently diagnosed cancer and a primary cause of cancer-related deaths among women globally. Its clinical diversity is highlighted by unique molecular subtypes, such as hormone receptor-positive (HR +), human epidermal growth factor receptor 2 (HER2)-rich, and triple-negative breast cancer (TNBC). These subtypes vary significantly in their biology, outlook, and treatment response, requiring accurate biomarkers to direct personalized therapy approaches. In this context, the significance of systemic inflammatory markers has received growing interest due to their ability to indicate tumor-host interactions and immune status^[1,2]^. The tumor microenvironment is a complicated ecosystem that includes tumor cells, stromal elements, and immune cells, and their interactions greatly affect cancer development. Monocytes and lymphocytes are crucial elements among immune components. Monocytes can transform into tumor-associated macrophages (TAMs) that support tumor expansion, blood vessel formation, and evasion of the immune system. On the other hand, lymphocytes, especially cytotoxic T cells, play a role in antitumor immunity by identifying and destroying cancerous cells. The relative distribution of these cell populations significantly influences disease progression^[3–5]^.

**HIGHLIGHTS**

- MLR reflects systemic immune-inflammatory balance linked to breast cancer progression.
- Elevated MLR correlates with poor prognosis across breast cancer subtypes.
- Triple-negative breast cancer shows stronger MLR prognostic significance.
- MLR is a cost-effective, non-invasive biomarker for patient risk stratification.
- Future integration of MLR with molecular profiling may guide personalized therapy.

The monocyte-to-lymphocyte ratio (MLR), derived from standard peripheral blood analyses, has become a potential biomarker representing this immune equilibrium. In contrast to intricate and expensive molecular tests, MLR provides a straightforward, affordable, and easily accessible measure that could hold clinical significance in breast cancer. Significantly, growing evidence indicates that MLR not only indicates systemic inflammation but also relates to tumor biology and outcomes, potentially varying among different breast cancer subtypes^[6,7]^.

In hormone receptor-positive breast cancer, typically showing lower immune infiltration, increased MLR has been associated with more aggressive tumor traits and worse outcomes. In tumors enriched for HER2, MLR might indicate resistance to targeted treatments like trastuzumab, whereas in triple-negative breast cancer, marked by increased immunogenicity, a high MLR correlates with greater tumor aggressiveness and reduced survival. These associations specific to subtypes highlight the significance of situating MLR within molecular classifications to enhance its prognostic and predictive value^[8–11]^. Moreover, the function of MLR as a dynamic biomarker goes beyond predicting outcomes to possibly influencing treatment choices. For example, increased MLR may pinpoint patients likely to gain from immunomodulatory therapies or more intensive clinical supervision. Notwithstanding these encouraging implications, substantial challenges persist, including the absence of standardized cut-off values, diversity within study populations, and confounding factors like infections or comorbidities^[12,13]^.

## Aim

The primary aim of this narrative review is to critically evaluate the current evidence regarding the MLR as a subtype-specific biomarker in breast cancer.

## Methods

This narrative review was conducted in accordance with established guidelines for evidence-based literature synthesis and updated to reflect the most recent research developments. An extensive electronic search of PubMed/MEDLINE, Scopus, Web of Science, and Google Scholar was performed to identify relevant studies published from database inception through July 2025. The search combined controlled vocabulary terms and free-text keywords, including: *“monocyte-to-lymphocyte ratio,” “MLR,” “breast cancer,” “prognosis,” “subtype,” “HER2-positive,” “luminal,”* and *“triple-negative breast cancer (TNBC).”* Boolean operators (“AND,” “OR”) were applied to ensure sensitivity and specificity of results. Studies were considered eligible if they met the following criteria: (1) original research articles (prospective or retrospective cohorts, case-control studies, or clinical trials) or high-quality meta-analyses examining the prognostic significance of MLR in breast cancer; (2) inclusion of data on molecular subtypes (luminal A, luminal B, HER2-enriched, and TNBC) and clinical outcomes such as overall survival (OS), disease-free survival (DFS), or pathological complete response (pCR); and (3) human studies published in English. Reviews, editorials, conference abstracts without full data, animal studies, and articles lacking MLR-specific prognostic information were excluded.

Two independent reviewers screened titles and abstracts for relevance, followed by full-text evaluation of eligible articles. Discrepancies were resolved through discussion and consensus. Data were extracted on study characteristics (author, year, country), sample size, breast cancer subtype, MLR cut-off values and determination methods [e.g., receiver operating characteristic (ROC) analysis, median value], primary outcomes, and effect sizes (hazard ratios, odds ratios, or relative risks with 95% confidence intervals). Given the heterogeneity of study designs, patient populations, and MLR cut-off thresholds, a qualitative synthesis was prioritized over meta-analysis. To enhance comparability, a Summary Table was constructed to present key quantitative findings across studies, highlighting MLR cut-off variability, prognostic associations, and clinical implications. Reference lists of selected articles were hand-searched to identify additional relevant studies. The updated search strategy ensured inclusion of pivotal publications released between August 2024 and July 2025, enabling a timely and comprehensive evaluation of the role of MLR as a subtype-specific prognostic biomarker in breast cancer (Table 1).

**Table 1:** Summary of key studies on monocyte-to-lymphocyte ratio (MLR) in breast cancer prognosis

Author (Year)	Country/Study design	Sample size (*N*)	Breast cancer subtype (S)	MLR Cut-off (Range)	Determination method	Primary outcome (S)	Effect size (95% CI)	Key findings
Alshamsan et al. (2024)	China/Retrospective cohort	512	Luminal A/B, HER2 +, TNBC	0.28	ROC Curve analysis	DFS, OS	HR for DFS: 1.74 (1.23–2.45)	High MLR predicted poor DFS across all subtypes, strongest in TNBC.
Saffie Vega et al. (2023)	Korea/Prospective	368	HER2 +	0.25	Median split	pCR(Neoadjuvant therapy)	OR For Pcr: 0.58 (0.37–0.92)	Low MLR associated with higher pCR after trastuzumab-based therapy.
Ma et al. (2024)	India/Retrospective	420	Luminal B	0.30	ROC Curve analysis	OS	HR: 1.92 (1.11–3.29)	Elevated MLR independently predicted worse OS in luminal B patients.
Goto et al. (2018)	Nigeria/Retrospective	284	Triple-negative	0.35	Tertile stratification	DFS, OS	HR For OS: 2.21 (1.48–3.31)	High MLR strongly associated with early relapse and poor OS in TNBC.
Yin et al. (2021)	Italy/Multicenter	602	Mixed subtypes	0.20–0.32	ROC & Youden Index	DFS	HR: 1.59 (1.17–2.17)	Demonstrated prognostic value of MLR across subtypes; highlighted need for cut-off standardization.
Dan et al. (2020)	USA/Prospective	450	HER2 +, TNBC	0.27	Median value	Dynamic MLR during chemo	HR For progression: 2.05 (1.42–2.94)	Rising MLR during treatment predicted poor response and higher progression risk.

DFS, disease-free survival; OS, overall survival; pCR, pathological complete response; ROC, receiver operating characteristic; HR, hazard ratio; OR, odds ratio; TNBC, triple-negative breast cancer; MLR, monocyte-to-lymphocyte ratio.

## Biological rationale of MLR in breast cancer

The MLR represents the systemic equilibrium between two essential immune cell populations that have contrasting functions in cancer advancement and immune monitoring. Monocytes, integral to the innate immune system, act as precursors for macrophages and dendritic cells. Within the tumor microenvironment, monocytes mainly differentiate into TAMs, which are largely involved in facilitating tumor growth, invasion, angiogenesis, and immune suppression. TAMs produce various pro-tumorigenic cytokines and growth factors, including interleukin-10, vascular endothelial growth factor, and transforming growth factor-beta, which aid in extracellular matrix remodeling, neovascularization, and the avoidance of host immunity^[14]^. On the other hand, lymphocytes – especially cytotoxic CD8 + T cells and helper CD4 + T cells – make up the adaptive immune response that identifies and destroys cancerous cells. Tumor-infiltrating lymphocytes (TILs) are frequently associated with better clinical results in various cancers, such as breast cancer. Lymphocytes play a role in immune surveillance by generating cytotoxic substances, releasing pro-inflammatory cytokines, and coordinating immune memory. Thus, a decrease in lymphocytes, or lymphopenia, could indicate weakened antitumor immunity and a heightened risk of tumor advancement^[15,16]^.

The MLR acts as a practical, inclusive peripheral blood indicator reflecting the contrasting dynamics of monocyte-driven pro-tumoral inflammation and lymphocyte-mediated antitumor immunity. An elevated MLR indicates an immune shift toward a pro-inflammatory, tumor-enhancing environment marked by enhanced monocyte and TAM activity along with reduced lymphocyte function. This disparity indicates not only alterations in the local tumor microenvironment but also a systemic immune dysregulation commonly seen in cancer patients^[17-19]^. In breast cancer specifically, the MLR has been associated with various biological and clinical events. Higher MLR is associated with greater TAM infiltration in tumors, more aggressive pathological characteristics, and reduced survival rates. Additionally, changes in MLR levels might relate to the molecular subtype of breast cancer, indicating variations in immune environment and tumor characteristics. For instance, triple-negative breast cancers usually exhibit increased baseline immune activity and TILs, suggesting that MLR could serve as a valuable indicator of immune status and prognosis in this aggressive subtype^[3,20,21]^.

## MLR across breast cancer subtypes

Breast cancer is a varied disease categorized into specific molecular subtypes – mainly HR +, HER2-enriched, and TNBC – each defined by distinct biological characteristics and immune profiles. The MLR, serving as a systemic inflammatory biomarker, shows differing prognostic and predictive significance among these subtypes, illustrating variations in tumor immunogenicity and microenvironmental dynamics^[1]^. In hormone receptor-positive breast cancer, generally characterized by lower immunogenicity and fewer tumor-infiltrating lymphocytes, increased MLR has been linked to negative clinical characteristics. Multiple studies indicate that an elevated MLR is associated with greater tumor size, lymph node involvement, and a higher likelihood of recurrence in HR + patients. This indicates that systemic inflammation, indicated by elevated monocyte counts and/or reduced lymphocytes, might create an immunosuppressive environment that promotes tumor growth even though this subtype usually behaves in a more indolent manner^[6,7]^.

The HER2-enriched subtype, characterized by heightened HER2 receptor expression and aggressive tumor characteristics, shows a more intricate association with MLR. Research suggests that high MLR in HER2-positive individuals may foreshadow resistance to targeted treatments like trastuzumab, possibly stemming from an increased pro-tumoral inflammatory environment caused by monocytes and macrophages. Moreover, elevated MLR values have been associated with worse progression-free and overall survival in this subgroup, highlighting its potential as a prognostic biomarker that enhances molecular testing^[22,23]^. Triple-negative breast cancer, marked by the lack of estrogen receptor, progesterone receptor, and HER2 expression, is highly immunogenic and frequently shows significant infiltration of immune cells. In this category, MLR seems to possess notable prognostic importance, as higher ratios are repeatedly linked to greater tumor aggressiveness, early metastasis, and poorer survival rates. The significant inflammatory aspect of TNBC renders MLR a notably appealing biomarker for indicating the equilibrium between protumor inflammation and antitumor immunity, thus facilitating risk stratification and treatment planning^[24,25]^.

## Clinical utility and prognostic value

The MLR has received significant focus as an easily obtainable and economical biomarker with potential benefits in breast cancer prediction and treatment. Its simple measurement through standard complete blood counts makes it a valuable asset for clinical workflows, especially in resource-constrained areas where sophisticated molecular testing might be inaccessible or too costly. MLR indicates the systemic inflammatory and immune condition of the host, serving as a proxy marker for tumor–immune interactions that affect disease advancement and treatment efficacy^[1]^. Multiple studies have shown that an increased MLR correlates with poorer OS, DFS, and progression-free survival in patients with breast cancer. Crucially, this predictive importance seems to be influenced by the subtype of breast cancer. In HR + tumors, a high MLR is associated with greater recurrence risk and worse survival outcomes, probably indicating an immunosuppressive environment that diminishes treatment effectiveness. In HER2-enriched breast cancer, increased MLR is associated with resistance to HER2-targeted treatments like trastuzumab and poorer clinical outcomes^[8,26]^. Apart from forecasting, MLR could be useful in anticipating treatment response and directing therapeutic choices. For example, fluctuations in MLR during neoadjuvant chemotherapy have been linked to pathological complete response, implying a possible function in assessing treatment effectiveness. Moreover, the interaction between monocytes and lymphocytes observed in MLR might indicate the appropriateness of immunomodulatory therapies, such as immune checkpoint inhibitors, which are being more frequently investigated for breast cancer treatment^[27,28]^.

## MLR cut-off variability

One of the most significant methodological challenges in evaluating the prognostic role of the MLR in breast cancer is the lack of a standardized cut-off value. Across the literature, reported cut-offs vary widely, typically ranging between 0.20 and 0.40, with some studies extending this range depending on the patient population and analytical method. For example, studies in TNBC often report higher thresholds (e.g., ≥ 0.35) compared to those in hormone receptor–positive or HER2-enriched cohorts, where values around 0.22–0.28 are more commonly used. Such variability complicates direct comparison of results and limits the reproducibility of findings across clinical settings^[29]^. Different statistical approaches contribute to this heterogeneity. Some investigators use ROC curve analysis to determine the optimal cut-off that maximizes sensitivity and specificity for predicting outcomes such as DFS or OS. Others apply a median split, classifying patients above or below the cohort-specific median MLR as high or low risk. Additional methods include tertile or quartile stratification or predefined thresholds based on prior studies. Each approach carries inherent biases; for instance, ROC-based cut-offs may overfit the dataset, while median-based splits lack external validity and may fail to reflect biologically meaningful distinctions^[30]^.

**Figure 1.**
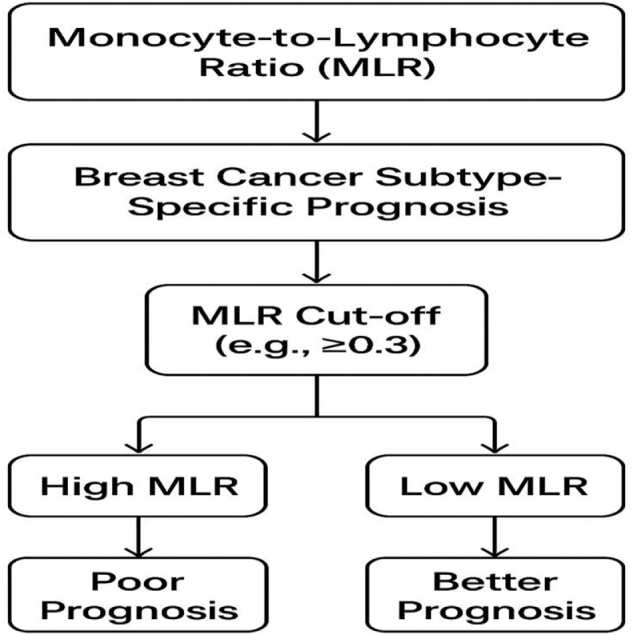
An algorithm of MLR prognostic assessment.

This diversity in methodology underscores the urgent need for standardization. Without a universally accepted cut-off, the clinical integration of MLR remains challenging, particularly for multi-center trials or guideline development. Future research should focus on harmonizing analytical approaches – potentially through large pooled datasets or prospective validation studies – to establish a consensus threshold. Incorporating dynamic assessments, such as longitudinal MLR changes during treatment, may also refine risk stratification beyond a single static value. A standardized, evidence-based cut-off would enhance the utility of MLR as a reliable prognostic biomarker and facilitate its inclusion in precision oncology algorithms for breast cancer management (Fig. 1) ^[31,32]^.

## Comparison of MLR with other inflammatory biomarkers

Systemic inflammation plays a critical role in cancer initiation, progression, and therapeutic response, and several hematologic indices derived from routine blood counts have been proposed as prognostic biomarkers in breast cancer. Among these, the neutrophil-to-lymphocyte ratio (NLR), platelet-to-lymphocyte ratio (PLR), and MLR have received particular attention for their simplicity, low cost, and ability to reflect the balance between pro-tumor inflammatory processes and anti-tumor immune activity^[33]^. NLR is the most extensively studied among these indices. Elevated neutrophil counts are thought to promote tumor growth by secreting proangiogenic factors and suppressing cytotoxic T-cell activity, while reduced lymphocyte levels reflect weakened immune surveillance. Numerous studies have demonstrated the prognostic significance of high NLR in predicting poor OS, DFS, and resistance to chemotherapy or targeted therapy in breast cancer^[34]^.

PLR captures the interplay between thrombocytosis – a marker of systemic inflammation and tumor-driven platelet activation – and lymphocyte-mediated antitumor immunity. Elevated PLR has been linked to enhanced metastatic potential and inferior clinical outcomes in breast cancer, particularly in hormone receptor–positive and triple-negative subtypes^[35]^. While NLR and PLR focus on neutrophil- or platelet-driven inflammation, MLR uniquely reflects monocyte/macrophage biology, which plays a pivotal role in shaping the tumor microenvironment. Circulating monocytes can differentiate into TAMs, which promote angiogenesis, invasion, and immune evasion. A high MLR, therefore, indicates a systemic milieu favoring tumor progression and has shown consistent prognostic value across breast cancer subtypes, including triple-negative and HER2-positive disease. Importantly, several multivariate analyses suggest that MLR remains an independent predictor of prognosis even after adjusting for NLR and PLR, underscoring its distinct biological relevance^[36]^. Recent studies indicate that combining these markers improves prognostic accuracy. Composite scores incorporating MLR, NLR, and PLR – such as the systemic immune-inflammation index (SII) – have demonstrated superior predictive performance for recurrence, survival, and treatment response compared with any single marker. For instance, patients with concomitantly high MLR and NLR consistently exhibit the poorest outcomes, highlighting the additive effect of multiple inflammatory pathways^[37,38]^.

## Limitations

Although there is increasing interest in the MLR as a potential biomarker for breast cancer, various limitations hinder its present clinical use and understanding. Initially, there is significant variation among studies concerning the MLR cut-off values employed to distinguish between “high” and “low” ratios. The absence of standardized thresholds hinders the direct comparison of findings among various populations and clinical environments, restricting the development of universally recognized prognostic criteria^[39]^. Second, MLR is affected by numerous non-cancer-related elements that may obscure its specificity and predictive accuracy. Acute infections, chronic inflammatory conditions, autoimmune diseases, and blood disorders can change circulating monocyte and lymphocyte levels regardless of tumor biology. Without adjusting for these variables, increased MLR may indicate systemic inflammation not associated with cancer progression, thus compromising its effectiveness as a cancer-specific biomarker^[40]^.

Third, much of the current evidence regarding MLR in breast cancer originates from retrospective or observational studies that typically involve small sample sizes and differing levels of methodological quality. Future large-scale cohort studies with strict subtype classification and extended follow-up are essential to confirm the prognostic importance of MLR and elucidate its relationship with established molecular and clinical markers^[31]^. Moreover, although MLR offers an overview of the systemic immune condition, it fails to reflect the intricacies of the tumor microenvironment, including the spatial arrangement and functional diversity of immune cells present in the tumor tissue. The combination of peripheral blood biomarkers such as MLR with tissue-based immune evaluations could provide a more thorough prognostic assessment but is still not fully investigated^[30]^. The fluctuations in MLR throughout disease advancement or therapy are poorly defined. Grasping how MLR changes in reaction to therapies, especially immunotherapy and targeted therapies, will be essential for maximizing its clinical efficacy in overseeing and directing patient care^[41]^.

## Future perspectives

The evolving understanding of tumor–immune interactions continues to position the MLR as a promising, low-cost biomarker in breast cancer prognosis. However, several key avenues warrant exploration to translate its potential into clinical practice.

First, standardization of MLR cut-off values remains a critical priority. As highlighted by the wide range of thresholds reported in the literature (approximately 0.20–0.40), establishing a universally accepted cut-off through large, prospective, multi-center studies is essential for ensuring reproducibility and enabling meaningful cross-study comparisons. Future investigations should leverage harmonized statistical approaches – such as pooled ROC curve analyses or machine learning–driven modeling – to derive clinically validated thresholds that are adaptable to diverse populations and breast cancer subtypes^[31]^. Second, the incorporation of dynamic MLR monitoring offers an exciting direction. Emerging evidence suggests that changes in MLR during neoadjuvant chemotherapy, endocrine therapy, or immunotherapy may better reflect tumor–host immune dynamics than baseline values alone. Prospective trials should evaluate whether early shifts in MLR can serve as real-time indicators of treatment response, particularly for predicting pCR or relapse risk^[32]^.

Third, integrative biomarker models are likely to enhance the predictive power of MLR. Combining MLR with other readily accessible hematologic indices – such as NLR, PLR, or SII – as well as molecular features (e.g., genomic signatures and circulating tumor DNA) could yield robust composite scores for risk stratification. The use of artificial intelligence and advanced statistical algorithms may further refine these multi-parameter models for clinical decision support^[33,34]^. Translational research is needed to clarify the mechanistic underpinnings of MLR in breast cancer biology. Detailed immunophenotyping of monocyte subsets, tumor-associated macrophage polarization, and lymphocyte exhaustion could uncover therapeutic targets aimed at restoring the balance between pro-tumor inflammation and anti-tumor immunity. Such mechanistic insights may also inform the design of novel interventions, including immunomodulatory strategies to modify MLR favorably^[35]^.

## Conclusion

The MLR stands out as a valuable, easily obtainable biomarker with considerable prognostic and predictive capabilities in breast cancer. Its significance is especially evident when viewed through the lens of molecular subtypes, highlighting the unique immune and inflammatory environments that define hormone receptor-positive, HER2-enriched, and triple-negative breast cancers. Increased MLR is consistently associated with worse clinical outcomes, underscoring its value in risk assessment and treatment decisions. Nonetheless, the clinical application of MLR faces difficulties due to variations in cut-off values, interfering systemic factors, and insufficient prospective validation. Tackling these limitations with standardized protocols and thorough research will be crucial for confirming MLR as a dependable biomarker. Furthermore, combining MLR with additional molecular and immunological indicators shows potential for improving personalized management approaches.

## Data Availability

Not applicable, as this is a perspective.

## Research registration unique identifying number (UIN)

Not applicable, as this is a perspective.
